# High microRNA-23a expression in laryngeal squamous cell carcinoma is associated with poor patient prognosis

**DOI:** 10.1186/s13000-015-0256-6

**Published:** 2015-04-03

**Authors:** Xiao-Wen Zhang, Ning Liu, Sheng Chen, Ye Wang, Zhao-Xiong Zhang, Yuan-Yuan Sun, Guang-Bin Qiu, Wei-Neng Fu

**Affiliations:** Department of Medical Genetics, China Medical University, No.77 Puhe Road, Shenyang North New Area, Shenyang, Liaoning Province 110122 P R China; Department of Laboratory Medicine, No. 202 Hospital of PLA, No.5, Guangrong Street, Heping district, Shenyang, Liaoning Province 110003 P. R. China

**Keywords:** MicroRNA-23a, Laryngeal squamous cell carcinoma, Prognosis, Biomarker

## Abstract

**Background:**

MicroRNA-23a (miR-23a) has been demonstrated to play an important role in the development of several types of cancer, but its role in tumorigenesis of laryngeal carcinoma is still unclear. The aim of this study was to investigate the expression patterns and clinical implications of miR-23a in laryngeal cancer.

**Methods:**

Quantitative RT-PCR was performed to evaluate the expression level of miR-23a in 52 pairs of laryngeal cancer. Analysis between miR-23a expression and clinical features of laryngeal carcinomas was performed by appropriate statistical methods. Role of miR-23a in laryngeal cancer cell migration and invasion was detected via transwell and matrigel assays, respectively.

**Results:**

miR-23a was significantly up-regulated in laryngeal cancer tissues compared to normal adjacent laryngeal tissues (*P* < 0.01). Tumors with high miR-23a expression had significantly greater extent of lymph node metastasis (*P* < 0.01), worse clinical stage (*P* < 0.05) and shorter overall five-year survival (*P* < 0.01) than those with low miR-23a expression. Both univariate and multivariate Cox hazard regression analysis results showed that clinical stage and miR-23a expression were significantly correlated with patient five-year survival (*P* < 0.01). miR-23a overexpression also significantly promoted laryngeal cancer cell migration and invasion in vitro.

**Conclusions:**

miR-23a, an independent prognostic factor for laryngeal cancer, participates in the onset and progression of laryngeal cancer.

**Virtual slides:**

The virtual slide(s) for this article can be found here: http://www.diagnosticpathology.diagnomx.eu/vs/2021488014982305

## Background

At present, the invasion and metastasis of tumor cells are considered as major causes of mortality in cancer patients [1]. According to the survey, the survival rate of laryngeal carcinoma patients with metastasis is only 30%–40% [2]. Although some tumor biomarkers such as carcinoembryonic antigen (CEA), carbohydrate antigen 19-9(CA19-9) are used to assess the possibility of tumors metastasis [3], there is still lack of ideal biomarkers for predicting invasion and metastasis of cancer patients.

MicroRNAs (miRNAs) are a category of small (19 ~ 24 nucleotides), non-coding and single-stranded endogenous RNA molecules that influence multiple biological events, especially the development and progress of malignancies [4-7]. Some studies have reported that miRNA could be used as a biomarker for the invasion and metastasis of several types of cancer [8-12]. However, the expression patterns and role of miRNAs in laryngeal carcinoma are seldom reported in the literature.

MiR-23a is aberrantly expressed in many cancers such as breast cancer, colorectal cancer, lung cancer, gastric cancer and glioma, and plays important roles in regulation of apoptosis and proliferation [13-18]. Similar to our recent work, miR-23a promotes proliferation and represses apoptosis in laryngeal squamou cell cancer (LSCC) (unpublished observations) and pancreatic cancer through directly targeting the APAF-1 3′UTR [19]. Also, miR-23a has been demonstrated to participate in regulation of invasion and metastasis in some cancers such as colorectal carcinoma and neuroblastoma [15,20]. Li et al. discovered a series of differentially expressed miRNAs including miRNA-23a in formalin-fixed paraffin-embedded laryngeal carcinoma tissues and the paired normal controls based on the miRNA microarray and found that miRNA-23a is associated with the lymphatic metastasis of laryngeal carcinoma [21]. However, the role of miR-23a in invasion and metastasis of laryngeal carcinoma is still unclear.

In the study, the miR-23a gene expression in laryngeal carcinoma tissues was detected by Quantitative RT-PCR. Correlations of miR-23a to survival and clinic-pathological features of LSCC patients were analyzed. Prognostic factors related to LSCC were revealed by univariate and multivariate analysis. Role of miR-23a in invasion and metastasis of Hep-2 (human laryngeal cancer) cells was also evaluated by matrigel and transwell assays, respectively.

## Methods

### Patients and tissue samples

This study was approved by the Research Ethics Committee of China Medical University (Shenyang, China) and the 463th Hospital of PLA (Shenyang, China). Written informed consent was obtained from all patients. All specimens were handled and made anonymous according to the ethical and legal standards.

Fifty-two pairs of laryngeal carcinoma tissue and paired adjacent normal tissue were obtained from each patient who underwent surgical resection treatment at the 463th Hospital of PLA (Shenyang, China) between 1999 and 2011, and were subsequently diagnosed based on histopathological evaluation. Normal laryngeal mucosa specimens were retrieved 10 mm outside the negative margin. Samples were placed in RNAlater Tissue Protect Tubes (Qiagen, Hilden, Germany) and stored at −80°C. Postsurgical pathology confirmed the diagnosis of squamous cell carcinoma in all patients. All patients did not receive radio- or chemotherapy before surgery.

### Cell culture and transfection

Hep-2 cell lines obtained from the Cell Biology Institute of Shanghai, Chinese Academy of Science were maintained in RPMI 1640 (GIBCO, Los Angeles, CA) medium supplemented with 10% fetal bovine serum (Hyclone, Logan, USA), 100 units/ml penicillin and 100 μg/ml streptomycin in a humidified atmosphere at 37°C with 5% CO_2_.

For transfection assay, Hep-2 cells were transfected with miR-23a inhibitor, inhibitor negative control, miR-23a mimic and miR-23a mimic negative control, respectively, by using Lipofectamine™2000 reagent (Invitrogen, Carlsbad, CA) according to the manufacturer’s instructions. All the nucleotide sequences used in the study are shown in Table 1.

**Table 1:** **The nucleotide sequences used in the study**

**Name**	**Sequence**
miR-23a mimics	5′-AUCACAUUGCCAGGGAUUUCC-3′
miR-23a inhibitor	5′-GGAAAUCCCUGGCAAUGUGAU-3′
mimics NC	5′-UUCUCCGAACGUGUCACGUTT-3′
inhibitor NC	5′-CAGUACUUUUGUGUAGUACAA-3′
NC	5′-GGCUACGUCCAGGAGCGCA CC-3′
miRNA-23a (reverse transcription primer)	5′-CTCAACTGGTGTCGTGGAGTCGGCAATTCAGTTGAGGGAAATCC-3′
miRNA-23a F	5′-ACACTCCAGCTGGGATCACATTGCCAGGGATTT-3′
miRNA-23a R	5′-TGGTGTCGTGGAGTCG-3′
U6F	5′-CTCGCTTCGGCAGCACA-3′
U6R	5′-AACGCTTCACGAATTTGCGT-3′
*GAPDH F*	5′-ATCATCAGCAATGCCTCC-3′
*GAPDH R*	5′-CATCACGCCACAGTTTCC-3′

Note: F and R indicates forward and reverse primers, respectively. NC shows the negative control.

### RNA extraction, reverse transcription and quantitative RT-PCR

Total RNA was extracted from the corresponding tissues using a miRcute miRNA isolation kit (Tiangen, Bejing, China) according to the manufacturer’s instructions. Reverse transcription was performed using the One Step Prime Script miRNA cDNA Synthesis Kit (Takara, Dalian, China) following the manufacturer’s instructions.

Quantitative RT-PCR was performed using SYBR® Premix Ex Taq™ II (Takara, Dalian, China) according to the manufacturer’s instructions with the 7500 real-time RT-PCR system (Applied Biosystems, Foster City). U6B was used as the normalization control. Each detection was performed in triplicate.

### Migration and invasion assays

Hep-2 Cells were suspended in serum-free medium. For migration and invasion assays, non-coated and Matrigel-coated membranes (24-well insert; 8-μm pore size; Corning Costar Corp) were used, respectively. The Matrigel-coated membrane was prepared with 30 μl Matrigel (BD Biosciences, San Jose, CA) and incubated for 40 min at 37°C in advance. In details, 1 × 10^5^ cells were plated in the top chamber with the non-coated and Matrigel-coated membranes, respectively. Medium supplemented with serum was used as a chemoattractant in the lower chamber. Cells were incubated for 24 hours at 37°C in a 5% CO_2_ incubator and those that did not migrate or invade through the pores were removed by a cotton swab. Cells on the lower surface of the membrane were then fixed with methanol, stained with hematoxylin and eosin, and subjected to microscopic inspection and photographed. Values for migration and invasion were obtained by counting five fields per membrane and each experiment was performed at least three times.

### Statistical analysis

SPSS16.0 software for Windows (SPSS Inc, IL, USA) was used for statistical analysis. Data were expressed as the mean ± standard deviation (SD). The differential expression of miR-23a between laryngeal cancer and the matched adjacent mucosa was evaluated by paired sample t-test. χ^2^ test was used to analyze association of miR-23a with survival. One-way ANOVO was applied to evaluate the relationship between miR-23a expression and the clinic-pathologic characteristics. Kaplan–Meier method was used for survival analysis. Difference in survival was estimated using the log-rank test. Risk factors for LSCC were examined by univariate and multivariate analyses (Cox proportional hazards regression model). *P <* 0.05 was considered statistically significant.

## Results

### Increased miR-23a expression participates in laryngeal carcinogenesis

**Figure 1:**
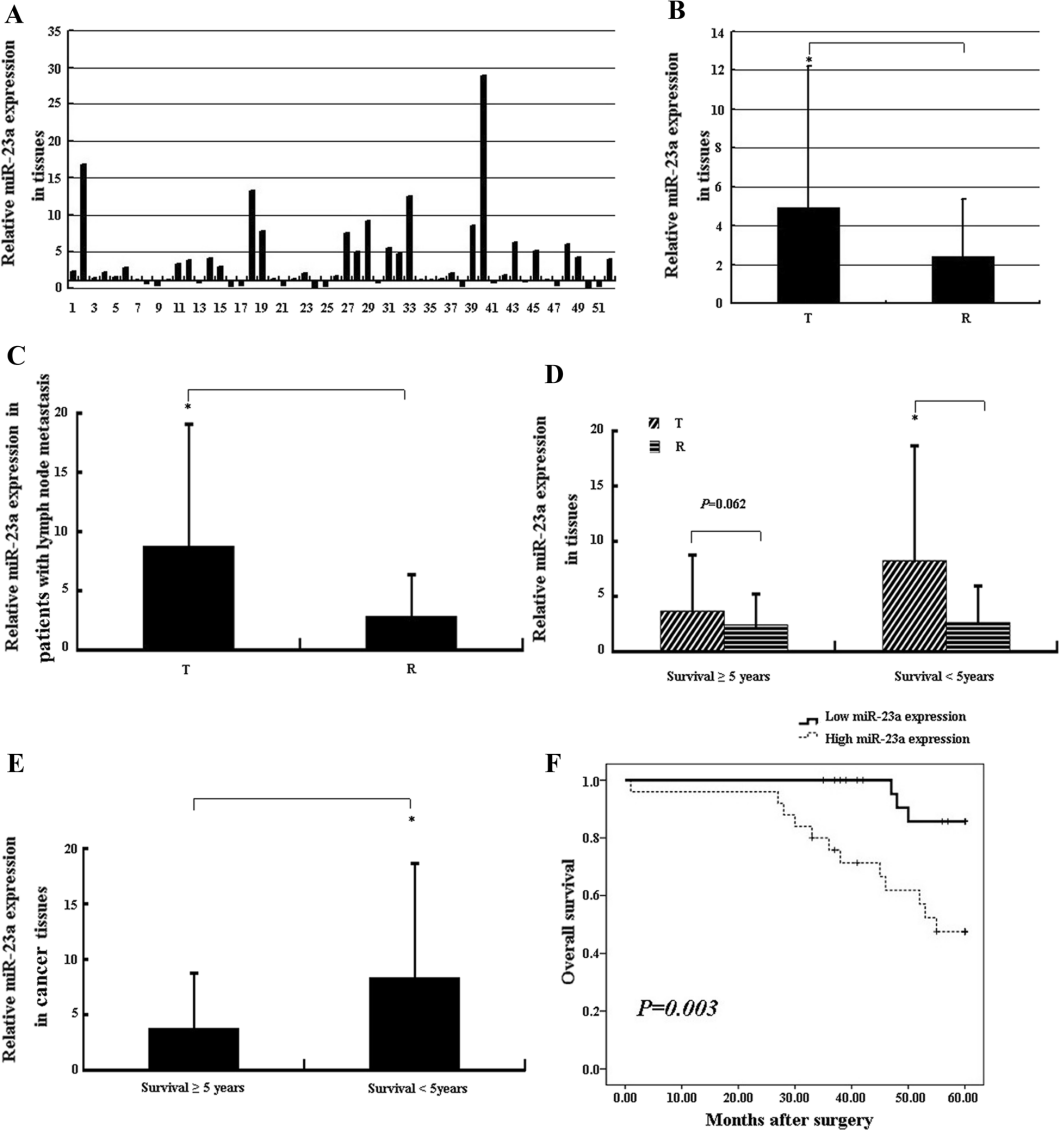
**miR-23a expression is associated with lymph node metastasis and survival rate in LSCC. (A)** Relative miR-23a expression in 52 LSCC patients. The relative expression was calculated as the ratio of miR-23a in cancer tissue to that in paired adjacent tissue in each case. The digit on the X-axis shows the number of samples used in the study. **(B)** General miR-23a expression in LSCC. T and R are cancer and matched adjacent tissues, respectively. miR-23a was normalized by U6 RNA. Symbol *indicates *P* < 0.05. **(C)** miR-23a expression in LSCC with lymph node metastasis. T and R are cancer and matched adjacent tissues, respectively. miR-23a was normalized by U6 RNA. *indicates *P* < 0.05, n = 18. **(D)** miR-23a expression in cancer and paired tissues in different survival groups. T and R are cancer and matched adjacent tissues, respectively. miR-23a was normalized by U6 RNA. *indicates *P* < 0.05, n = 37 in survival less than 5-year group and n = 15 in survival more than 5-year group. **(E)** miR-23a expression in cancer tissues in different survival groups. miR-23a was normalized by U6 RNA. *indicates *P* < 0.05, n = 52. **(F)** Postoperative 5-year survival curves of LSCC patients correlated to miR-23a expression. Kaplan–Meier estimates of overall 5-year survival for the LSCC patients with low miR-23a expression (fold change ≤ 2) and high miR-23a expression (fold change >2), respectively. The *P* value was calculated using the Log-rank test between patients with low and high miR-23a expression.

miR-23a expression was detected in 52 pairs of laryngeal cancer tissue and the matched adjacent tissue by qRT-PCR. As showed in Figure 1A, miR-23a level was increased in 37 out of 52 (71.2%) laryngeal cancer tissues compared to the controls. In general, miR-23a was significantly up-regulated in laryngeal cancer tissues compared with normal counterpart (*P* < 0.05) (Figure 1B), suggesting that miR-23a takes part in the genesis of laryngeal cancer.

### High miR-23a level contributes to poor prognosis in patients with laryngeal carcinomas

In 18 cases of patients with lymph nodes metastasis, miR-23a was significantly up-regulated in laryngeal cancer tissues compared to the normal counterpart (*P* < 0.05) (Figure 1C; Table 2). In patients aged less than 5 years after operation, miR-23a expression level was significantly higher in cancer tissues than that in controls (*P* < 0.05) (Figure 1D). miR-23a average level in cancer tissues was at higher level in patients aged less than 5 years, which showed a significant difference compared to that in patients aged more than 5 years (*P* < 0.05) (Figure 1E). These results imply that miR-23a expression level correlates with lymph nodes metastasis and patients’ survival in laryngeal cancer.

**Table 2:** **Association between miR-23a and clinicopathological characteristics in 52 patients with LSCC**

**Features**	**No.of cases**	**miR-23a expression**	***P*** **-value**
Age			
<60	22	3.87 ± 4.50	0.234
≥60	30	6.35 ± 10.00	
Gender			
male	45	5.17 ± 7.85	0.543
female	7	3.32 ± 2.27	
Smoking			
Nonsmokers	7	3.48 ± 5.36	0.584
Current smokers	45	5.14 ± 7.65	
Drinking			
drinker	37	5.54 ± 8.31	0.346
nondrinker	15	3.39 ± 4.08	
Differentiation			
well	15	3.06 ± 2.25	0.251
Moderate	29	4.91 ± 8.43	
Poor	8	8.46 ± 8.88	
Lymph node			
Negative	34	2.93 ± 3.72	0.006*
Positive	18	8.67 ± 10.63	
Tumor depth (pT)			
T1	9	2.52 ± 2.17	0.226
T2	15	3.37 ± 5.95	
T3	15	4.97 ± 5.77	
T4	13	8.31 ± 11.29	
Clinical stage			
I	6	2.04 ± 1.67	0.011*
II	12	1.88 ± 1.67	
III	31	5.54 ± 6.6	
IV	3	16.44 ± 20.48	

One-way ANOVO was used to analyze the correlation between the expression of miR-23a and clinicopathological features of the patients. *indicates *P* <0.05.

To better understand the potential roles of miR-23a in LSCC development and progression, one-way ANOVO was used to determine the relationships of miR-23a with various clinical features of LSCC, including age, gender, smoking, drinking, lymph nodes metastasis, differentiation, and clinic-pathological stage. As shown in Table 2, miR-23a overexpression in tumor tissues was strongly associated with lymph node metastasis and advanced clinical stage of LSCC (*P* = 0.006 and *P* = 0.011, respectively).

We then used Kaplan–Meier method, log-rank test and Cox proportional hazard regression model to analyze the survival correlation of LSCC patients with miR-23a expression and clinic-pathological features. Up-regulated more than 2-fold is considered to be high miR-23a expression. As indicated in Figure 1F, high miR-23a level was correlated with shorter overall five-year survival of LSCC patients (log-rank test: *P* = 0.003). Univariate Cox hazard regression analysis results showed that LSCC patient survival was significantly correlated with tumor depth, lymph node metastasis, clinical stage and miR-23a expression. However, only clinical stage and miR-23a expression were significantly correlated with patient survival after multivariate analysis was performed (Table 3).

**Table 3:** **Univariate and multivariate Cox hazard regression analysis for prognostic factors**

	**Univariate analysis**		**Multivariate analysis**
	**Hazard ratio (95% confidence interval)**	***P*** **value**	**Hazard ratio (95% confidence interval)**
**Gender**			
Male vs female	1.349(0.458-3.973)	0.587	
**Age**			
≥60 vs <60	1.849(0.760-4.498)	0.176	
**Smoking**			
Smoker vs Nonsmoker	0.848(0.250-2.868)	0.790	
**Drinking**			
Drinker vs nondrinker	0.818(0.345-1.942)	0.649	
**Differentiation**			
Poor vs moderate vs well	0.762(0.415-1.399)	0.381	
**Tumor depth (PT)**			
T4vs T3 vs T2 vs T1	2.173(1.380-3.422)	0.001*	
**Lymph node metastasis**			
Positive vs negative	4.243(1.844-9.764)	0.001*	
**Clinical stage**			
IVvs IIIvs IIvsI	5.513(2.300-13.214)	*P* < 0.001*	6.998(2.217-22.083)
**miR-23a expression**			
High vs low	7.419(2.561-21.491)	*P* < 0.001*	6.712(2.076-21.700)

*indicates*P* < 0.05.

### miR-23a promotes cell migration and invasion in laryngeal cancer Hep-2 cells

**Figure 2:**
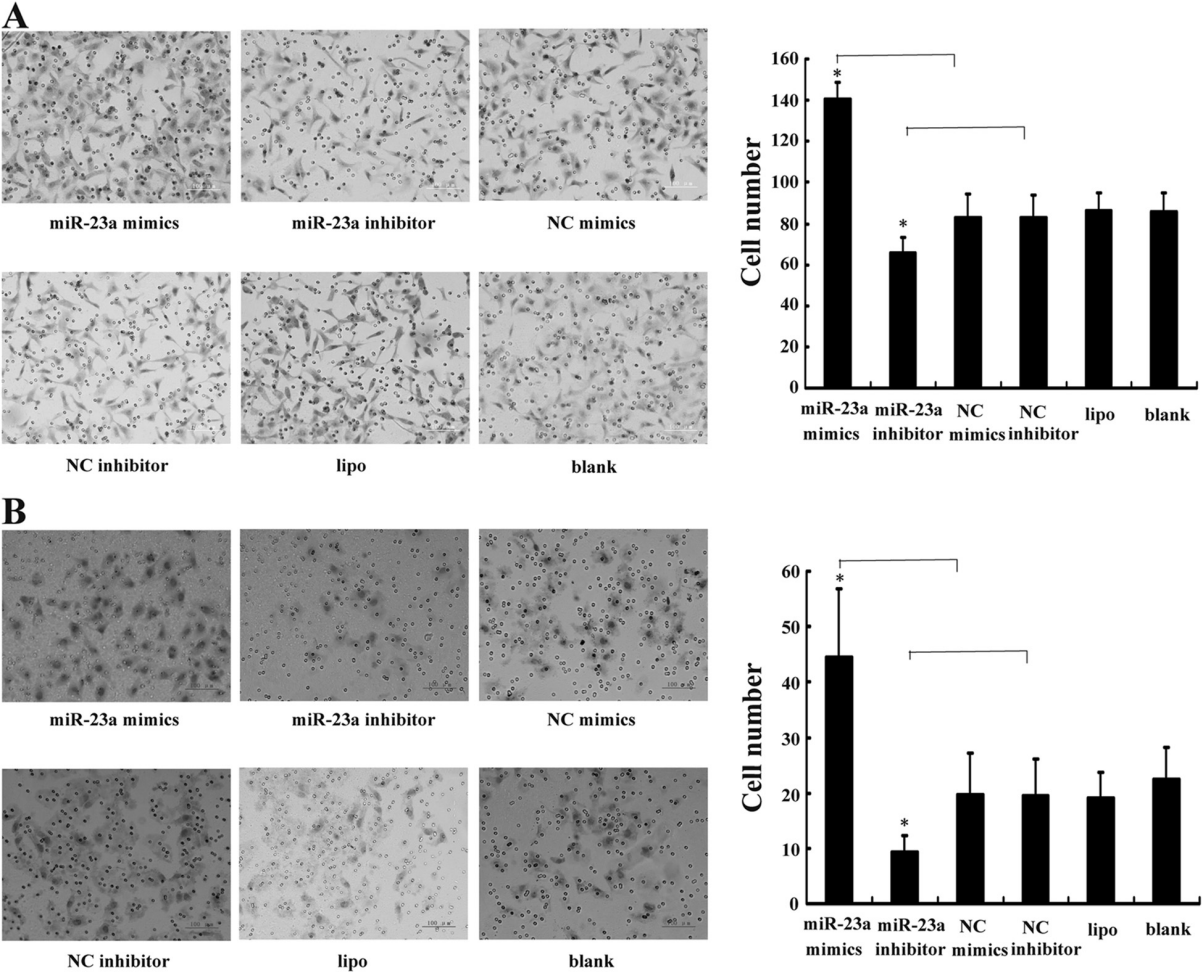
**miR-23a promotes the Hep-2 cell migration and invasion. (A)** Representative fields of migrated cells on the underside of membrane. **(B)** Representative fields of invasive cells on the underside of membrane. Data are presented as mean ± SD of at least three independent experiments. *indicates *P* <0.05.

To confirm the role of miR-23ain laryngeal cancer metastasis, we examined the effect of miR-23aon laryngeal cancer cell migration and invasion in vitro. As results, both migrated and invaded cells significantly increased and decreased in miR-23a mimic and miR-23a inhibitor groups compared to the controls, respectively, implying that miR-23a promotes laryngeal cancer cell migration and invasion (Figure 2).

## Discussion

Several genes, such as *P53*, *HER2* and *Bcl-2*, are considered to be tissue biomarkers for the early diagnosis of malignant tumor or assessing the prognosis of patients with tumor [22,23]. However, their clinical utility is limited due to their inexistence in body fluids. It is surprising that miRNAs are easily detected by qRT-PCR [24,25]. Moreover, miRNAs are found stable in 12 different biological fluids such as plasma, saliva, tears, urine, amniotic fluid, colostrum, breast milk, bronchial secretions, cerebrospinal fluid, peritoneal fluid, pleural fluid and seminal fluid [26]. Furthermore, these circulating miRNAs from different fluids especially from serum and urine can reflect the state of a given disease, suggesting that they are useful biomarkers in a broad range of clinical applications in human diseases [27].

In the study, we found miR-23a was significantly up-regulated in laryngeal carcinoma tissues compared to the adjacent tissues, which is consistent with previous findings in most solid tumors such as colon carcinoma, lung cancer and neuroblastoma, respectively [15,16,20]. In contrast, miR-23a is found to be down-regulated in chronic or acute leukemia [10,28] , respectively. Nevertheless, the abnormal miR-23a expression plays a critical role in carcinogenesis.

In addition, we found that the miR-23a gene expression in laryngeal carcinoma patients with lymph node metastasis is significantly higher than that in patients without lymph node metastasis. The result is consistent with previous report [21]. Our results also indicated that high level of miR-23a correlates with LSCC clinical stages, suggesting that miR-23a participates in laryngeal cancer cell migration and invasion, which is confirmed in vitro in the study. These results are in line with previous findings in colorectal cancer and neuroblastoma [15-20].

The overall five-year survival of laryngeal cancer patients has not improved (at approximately 35–70%). Conversely, it decreases in the United state [29,30]_._ Therefore, there is a great need to identify new biomarkers for the diagnosis and prognosis of laryngeal carcinoma. Our present study demonstrated that high miR-23a level in laryngeal cancer tissues significantly correlates with poorer five-year survival, which is reconfirmed by our multivariate Cox hazard regression analysis, implying that miR-23a expression level is an independent prognostic factor for laryngeal cancer in addition to clinical stage. Similar result is also found in liver cancer [31]. In addition to miR-23a, we also found that clinical stage is also related to poor progression in LSCC.

In the future, our work will focus on the molecular mechanism of miR-23a in laryngeal cancer cell migration and invasion.

## Conclusion

miR-23a is an independent prognostic factor for laryngeal cancer and participates in the onset and progression of laryngeal cancer.

## Abbreviations

LSCC: Laryngeal squamou cell cancer
miRNA: microRNA
UTR: Untranslation region
